# Regulation of Lipogenesis by Glucocorticoids and Insulin in Human Adipose Tissue

**DOI:** 10.1371/journal.pone.0026223

**Published:** 2011-10-14

**Authors:** Laura L. Gathercole, Stuart A. Morgan, Iwona J. Bujalska, David Hauton, Paul M. Stewart, Jeremy W. Tomlinson

**Affiliations:** Centre for Endocrinology, Diabetes and Metabolism, Institute of Biomedical Research, School of Clinical and Experimental Medicine, University of Birmingham, Birmingham, United Kingdom; International Centre for Genetic Engineering and Biotechnology, Italy

## Abstract

Patients with glucocorticoid (GC) excess, Cushing's syndrome, develop a classic phenotype characterized by central obesity and insulin resistance. GCs are known to increase the release of fatty acids from adipose, by stimulating lipolysis, however, the impact of GCs on the processes that regulate lipid accumulation has not been explored. Intracellular levels of active GC are dependent upon the activity of 11β-Hydroxysteroid dehydrogenase type 1 (11β-HSD1) and we have hypothesized that 11β-HSD1 activity can regulate lipid homeostasis in human adipose tissue (Chub-S7 cell line and primary cultures of human subcutaneous (sc) and omental (om) adipocytes. Across adipocyte differentiation, lipogenesis increased whilst β-oxidation decreased. GC treatment decreased lipogenesis but did not alter rates of β-oxidation in Chub-S7 cells, whilst insulin increased lipogenesis in all adipocyte cell models. Low dose Dexamethasone pre-treatment (5 nM) of Chub-S7 cells augmented the ability of insulin to stimulate lipogenesis and there was no evidence of adipose tissue insulin resistance in primary sc cells. Both cortisol and cortisone decreased lipogenesis; selective 11β-HSD1 inhibition completely abolished cortisone-mediated repression of lipogenesis. GCs have potent actions upon lipid homeostasis and these effects are dependent upon interactions with insulin. These *in vitro* data suggest that manipulation of GC availability through selective 11β-HSD1 inhibition modifies lipid homeostasis in human adipocytes.

## Introduction

The magnitude of the obesity epidemic has heightened the urgency to understand the mechanisms that contribute to the regulation of adipose tissue mass. The morbidities associated with obesity, especially central obesity, are well described and include dyslipidaemia, insulin resistance, type 2 diabetes (T2DM), hypertension and cardiovascular disease, all of which contribute to increased mortality [1].

The regulation of adipose tissue mass is complex. Increases in adipose tissue arise as a consequence of both hyperplasia (increase in fat cell number) and hypertrophy (increase in fat cell size) [2]. Hyperplasia relies on pre-adipocyte proliferation and differentiation into mature adipocytes. Adipocyte differentiation is a tightly regulated process orchestrated by the temporal expression of key transcription factors resulting in cyto-skeletal changes as well as the induction of key genes involved in lipid metabolism, namely lipogenesis, fatty acid uptake, lipolysis and β-oxidation. In mature adipocytes the relative balance of these processes is crucial in determining adipocyte size (and hence fat mass). Increased lipogenesis and fatty acid uptake favour lipid accumulation within the adipocyte, whilst lipolysis and β-oxidation promote lipid loss.

Lipogenesis occurs either as a consequence of re-esterification of free fatty acids (FFA) with glycerol or *de novo* synthesis of triacylglycerol (TAG). *De novo* lipogenesis may account for up to 20% of lipid turnover within adipose tissue [3]. During *de novo* lipogenesis, acetyl CoA is carboxylated to malonyl-CoA by acetyl CoA carboxylase (ACC) which is subsequently converted by a multi-step reaction to palmitate by fatty acid synthase (FAS) [4]. There are two isoforms of ACC (ACC1 and ACC2); in lipogenic tissues ACC1 predominates and is the key regulatory step of fatty acid synthesis. ACC2 is localized to the mitochondrial membrane, and its role is to limit β-oxidation through malonyl-CoA mediated inhibition of carnitine palmitoyl transferase I (CPT I) which catalyses the transfer of long-chain fatty acyl-CoA into the mitochondrion. ACC1 and 2 are highly regulated at the level of mRNA expression, protein phosphorylation and also by ubiquitination and proteasomal degradation. Phosphorylation by AMP-kinase (AMPK) at ser-79 on ACC1 and ser-218 on ACC2, negatively regulates activity [5].

Patients with glucocorticoid (GC) excess, Cushing's syndrome, develop a classical phenotype characterized by insulin resistance, proximal myopathy and central obesity. Whilst it is clear that GCs are essential for adipocyte differentiation [6], their impact upon many of the processes that regulate lipid accumulation within the adipocyte have not been explored in detail. *In vivo* and *in vitro*, GCs appear to stimulate lipolysis through a putative action upon hormone sensitive lipase [7], [8] and the resultant generation of FFA may contribute to the development of insulin resistance [9]. This is consistent with their role as ‘stress’ hormones, mobilizing FFA for β-oxidation, but a potential impact upon ACC activity and lipogenesis and β-oxidation has not been investigated. This may be important in explaining the clinical impact of endogenous and exogenous GC excess.

There has been much interest in the role of endogenous GC secretion and metabolism in the pathogenesis of human obesity. GC availability and action depend not only upon circulating levels but also on tissue specific intracellular activation or inactivation by 11β-hydroxysteroid dehydrogenases (11β-HSD). 11β-HSD1 is highly expressed in adipose tissue and predominantly converts inactive cortisone to cortisol, thus amplifying local GC action independent of circulating cortisol concentrations [10]. Within subcutaneous (sc) adipose tissue, there is increased 11β-HSD1 expression and activity that positively correlates with obesity and insulin resistance [11], [12]. Furthermore, studies employing 11β-HSD1 inhibitors have demonstrated clinical efficacy in improving insulin sensitivity in patients with type 2 diabetes [13].

Based upon the hypothesis that GC-mediated changes in lipid homeostasis in adipose tissue are a crucial driver to the Cushing's phenotype, we have performed a detailed characterization of the impact of GC on lipid flux in human adipose tissue. Furthermore we have proposed that the action of selective 11β-HSD1 inhibitors to improve insulin sensitivity may be in part medicated by suppression of lipid mobilization from adipose tissue decreasing FFA availability.

## Methods

### Cell Culture

#### Chub-S7 cell culture

The Chub-S7 cell line is a transformed human subcutaneous pre-adipocyte cell line derived from an obese female that has been validated as a good model of human adipocyte biology [14], [15]. Proliferating cells were cultured in Dulbecco's MEM/Nutrient Mixture F12, DMEM-F12 (Sigma, Poole, UK) with 10% foetal calf serum. At confluence, cells were differentiated for 14 days into mature adipocytes in chemically defined media (DMEM-F12 media with Biotin 33 µM, Pantothenate 17 µM, T_3_ 0.2 nM, Insulin 167 nM, Cortisol 1 µM and Rosiglitazone 1 µM).

#### Primary subcutaneous and omental pre-adipocyte cell culture

Paired primary human subcutaneous and omental pre-adipocytes were obtained from ZenBio (Durham, US) and were isolated from adipose tissue of healthy, non-diabetic donors aged 18–60 years undergoing elective surgery. Pre-adipocytes were cultured to confluence as per the manufacturer's guidelines using the supplied media. Once confluent, media were changed to a chemically defined media (ZenBio) and were differentiated into adipocytes for 14 d.

### RNA extraction and Reverse Transcription

Total RNA was extracted from cells using the Tri-Reagent system. RNA integrity was assessed by electrophoresis on 1% agarose gel. Concentration was determined spectrophotometrically at OD_260_. In a 50 µl volume 500 ng of total RNA was incubated with 250 µM random hexamers, 500 µM dNTPs, 20 U RNase inhibitor, 63 U Multiscribe reverse transcriptase, 5.5 mM MgCl and 1× reaction buffer (Applied Biosystems, Foster City, CA, USA). The reverse transcription reaction was carried out at 25°C for 10 min, 48°C for 30 min and then the reaction was terminated by heating to 95°C for 5 min.

### Real-Time PCR

mRNA levels were determined using an ABI 7500 sequence detection system (Perkin-Elmer Applied Biosystems, Warrington, UK). Reactions were performed in 10 µl volumes on 96-well plates in reaction buffer containing 2× TaqMan Universal PCR Master mix (Applied Biosystems). All primers and probes were supplied by applied biosystems ‘assay on demand’ and reactions normalised against the house keeping gene 18S rRNA, provided as a preoptimized control probe. All target genes were labelled with FAM and the housekeeping gene with VIC. The reaction conditions were as follows: 95°C for 10-minutes, then 40 cycles of 95°C for 15-seconds and 60°C for 1 min.

Data were obtained as ct values (ct = cycle number at which logarithmic PCR plots cross a calculated threshold line) and used to determine Δct values (Δct = (ct of the target gene)−(ct of the housekeeping gene). Data were expressed as arbitrary units using the following transformation [expression = 1000_*_(2^−Δct^) arbitrary units (AU)].

### Protein extraction and Immunoblotting

Total protein was extracted from cells using RIPA buffer (50 mM Tris pH 7.4, 1% NP40, 0.25% sodium deoxycholate, 150 mM NaCl, 1 mM EDTA, 1 mM PMSF and protease inhibitor cocktail (Roche, Lewes, UK) dissolved in 10 mL of distilled water) and freeze thawing. Protein concentrations were measured using a commercially available assay (Bio-Rad Laboratories Inc., Hercules, CA). 15 µg of protein was resolved on a 12.5% SDS PAGE gel and transferred onto nitrocellulose membrane, Hybond ECL (GE Healthcare, Chalfont St Giles, UK). Primary (anti-AMPK, pAMPK(Thr172), ACC1/2 and pACC(ser79/218), (Cell Signalling Technology, Danvers, MA) and secondary antibodies (Dako, Glostrop, UK) were used at a dilution of 1/1000. Membranes were re-probed for β-Actin. Primary and secondary antibodies were used at a dilution of 1/5000 (Abcam plc, Cambridge, UK). Bands were quantified with Genesnap by Syngene (Cambridge, UK) and expressed relative to β-actin to normalise for gel loading.

### Lipogenesis

Lipogenesis was measured by the uptake of 1-[^14^C]-acetate into the lipid component as described previously [16]. At confluence or after 14 days of differentiation Chub-S7 cells, or primary sc and om cells were washed and cultured for 4 h in serum free media. Cells were then incubated in 500 µl of serum free media with or without insulin (5 nM), glucorticoids (5 or 500 nM) and the selective 11β-HSD1 inhibitor (PF-877423, 5 µM, provided through a material transfer agreement with Pfizer inc. (La Jolla, CA, USA) [17]) for 18 h, 0.12 µCi/L 1-[^14^C]-acetic acid with cold sodium acetate [10 µM] was added to the treatments for a further 6 h. After incubation cells were washed and scraped into 250 µl of PBS. The lipid fraction was recovered in Folch solvent, the solvent was evaporated and the radioactivity retained in the cellular lipid was determined by scintillation counting and expressed as disintegrations per minute (dpm)/per well.

### β-oxidation

Rates of β-oxidation were measured by the conversion of [^3^H]-palmitate to [^3^H]-H_2_O. At confluence or after 14 days of differentiation, Chub-S7 cells were washed and cultured in serum free media for 4 h. Cells were then incubated with 300 ul of low glucose serum free media with 0.12 µCi/L [^3^H]-palmitic acid with cold palmitate to a final concentration of 10 µM palmitate and treated with or without insulin (5 nM) or Dex (5 or 500 nM) for 24 h. After incubation medium was recovered and precipitated with an equal volume of 10% tricholoroacetic acid. The aqueous component of the supernatants was extracted with 2∶1 choloform methanol solution. Radioactivity was determined by scintillation counting and expressed as disintegrations per minute (dpm)/per well.

### Fatty acid uptake

Fatty acid uptake was measured by intracellular accumulation of [^3^H]-palmitate. At confluence or after 14 days of differentiation, Chub-S7 cells were washed and cultured in serum free media for 4 h. Cells were then incubated with 300 ul of low glucose serum free media with 0.12 µCi/L [^3^H]-palmitic acid with cold palmitate to a final concentration of 10 µM palmitate and treated with or without Dex (5 or 500 nM) for 24 h. The cells were incubated at 37°C for 24 h. After incubation cell lysate was recovered and radioactivity was determined by scintillation counting and expressed as disintegrations per minute (dpm)/per well.

### Statistical analysis

Data are presented as mean ± standard error. Where data were normally distributed, t-tests (paired or unpaired where appropriate) were used to compare single treatments to control. If normality tests failed, non-parametric tests were used. ANOVA was used to compare multiple doses and/or treatments. (SigmaStat 3.1, Systat Software, Inc. Point Richmond, CA). Statistical analysis on real-time PCR data was performed on mean Δct values and not fold changes.

## Results

### Lipid metabolism across human adipocyte differentiation

**Figure 1:**
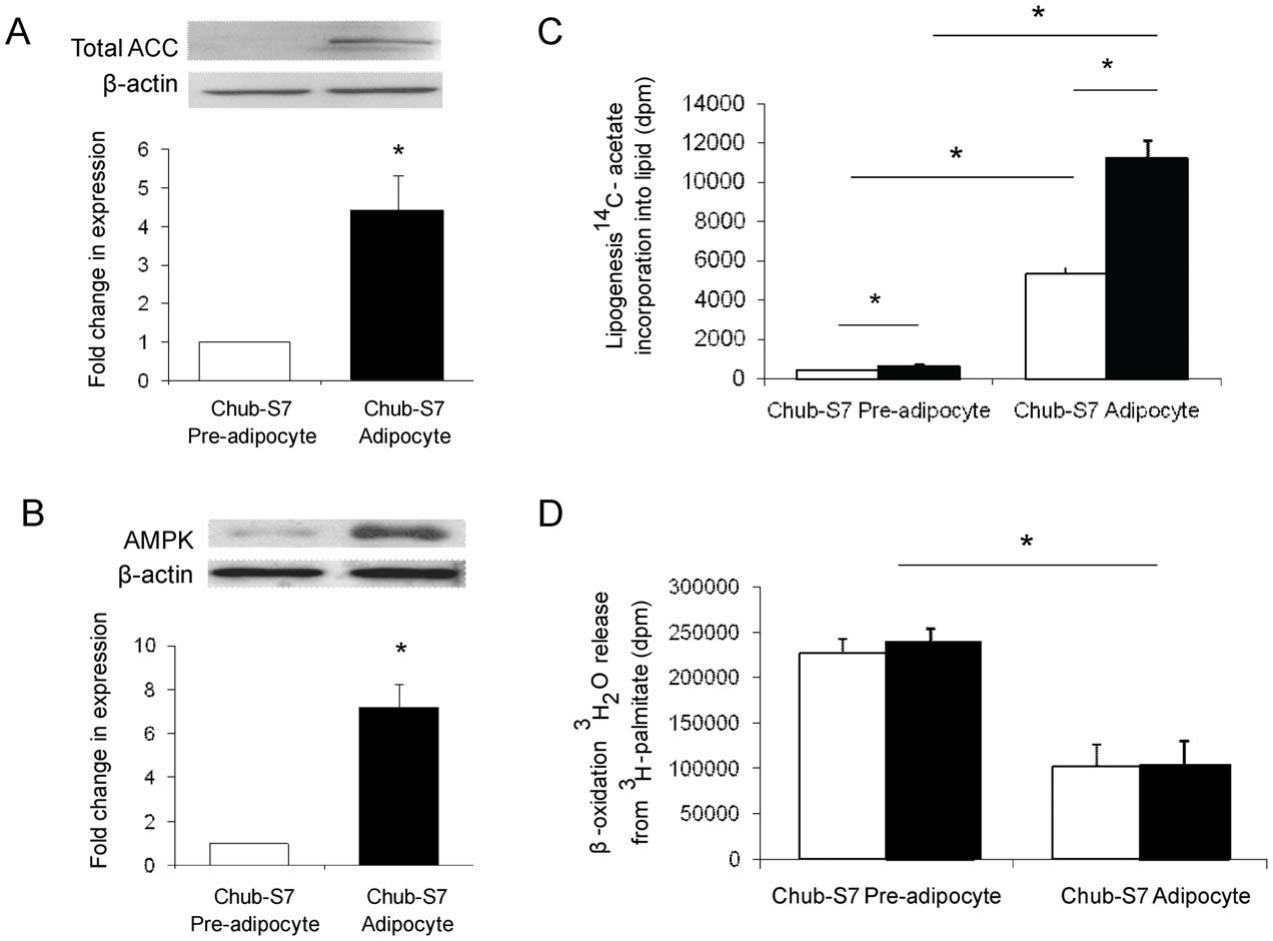
Across Chub-S7 differentiation, total ACC (a) and AMPK (b) levels increase. Data are presented as the mean±se fold change compared to undifferentiated cells and quantified relative to β-actin with representative western blots above (n = 3–5 experiments, * p<0.05). 1-[^14^C]-acetate incorporation into lipid increased across differentiation and was stimulated by insulin in both undifferentiated pre-adipocytes and differentiated adipocytes (c). In contrast, ^3^H release from ^3^H-palmitate decreased across differentiation and was not regulated by insulin. Data are expressed as disintegrations per minute (dpm) and are the mean±se of n≥3 experiments performed in triplicate. Basal (open bars) and insulin stimulated (black bars) and *p<0.05.

Across differentiation, mRNA expression levels of genes involved in lipid metabolism increased significantly in Chub-S7 cells (FAS 13.6-fold p<0.01; ACC1 2.2-fold p<0.05; ACC2 4.5-fold p<0.005). This was comparable to human cultures of primary sc pre-adipocytes (FAS 3.6-fold p<0.05, ACC2 24.1-fold p<0.05) although in primary sc cultures, ACC1 expression did not increase significantly across adipocyte differentiation (table 1). mRNA expression of AMPKα1 or AMPKα2 did not change across differentiation in Chub-S7 or sc pre-adipocytes. Endorsing our findings at the mRNA level, ACC1/2 protein expression increased in Chub-S7 cells (data expressed relative to undifferentiated cells, 1.0 vs. 4.3±1.8, p<0.05) (figure 1a). Although AMPK mRNA expression did not change, protein levels increased (1.0 vs. 7.2±1.8, p<0.001) (figure 1b).

**Table 1:** mRNA expression levels in primary cultures of undifferentiated (day 1) and differentiated (day 14) pre-adipocytes from Chub-S7 cells and subcutaneous pre-adipocytes tissue as measured by real-time PCR.

	Chub-S7 cells			Subcutaneous Pre-adipocytes		
Gene	Pre-adipocytes (day 1)	Differentiated (day 14)	p	Pre-adipocytes (day 1)	Differentiated (day 14)	p
**FABP4**	0.01±0.003	75.5.±6.79	<0.005	1.46±0.41	37.57±11.86	<0.005
**FAS**	0.57±0.05	7.15±0.9	<0.01	1.23±0.23	6.54±1.39	<0.005
**ACC1**	0.35±0.04	0.79±0.05	<0.05	0.28±0.03	0.54±0.09	ns
**ACC2**	0.05±0.01	2.03±0.14	<0.005	0.07±0.04	2.88±0.74	<0.005
**AMPKα1**	0.63±0.07	0.41±0.02	ns	0.88±0.53	0.70±0.24	ns
**AMPKα2**	0.07±0.01	0.04±0.004	ns	0.05±0.02	0.09±0.05	ns
**COP1**	0.56±0.04	0.48±0.04	ns	0.48±0.12	0.28±0.08	ns
**TRB3**	0.41±0.13	0.13±0.01	ns	0.94±0.26	0.16±0.01	<0.05

Data are presented as arbitrary units (mean±se, n = 3–6 experiments).

Consistent with an increase in the expression of genes involved in lipid metabolism 1-[^14^C]-acetate incorporation into lipid was higher in differentiated Chub-S7 adipocytes compared to undifferentiated cells (412±44 vs. 5351±304 dpm/well, p<0.05) (figure 1c). Furthermore, insulin-stimulated 1-[^14^C]-acetate incorporation into lipid in both undifferentiated and differentiated cells (undifferentiated: 412±44 vs. 662±93 dpm/well, p<0.05; differentiated: 5351±304 vs. 11250±880 dpm/well, p<0.05) (figure 1c). In contrast, [^3^H]-palmitate breakdown into water, reflecting β-oxidation, decreased across differentiation (227058±15548 vs. 101687±24079 dpm/well, p<0.05). Insulin treatment did not impact β-oxidation in either differentiated or undifferentiated cells (undifferentiated: 227058±15548 vs. 238677±16261 dpm/well, p = ns; differentiated: 101687±24079 vs. 100251±29916 dpm/well, p = ns) (figure 1d).

### Glucocorticoid and insulin regulation of lipid metabolism in human adipocytes

#### Lipogenesis

In Chub-S7 cells high (500 nM, 24 h), but not low (5 nM, 24 h) dose Dex, decreased ACC1 mRNA expression, but increased expression of FAS and ACC2 (table 2a and b). Although it did not reach significance Dex increased expression of FAS and ACC2 in sc, but not om cells (FAS sc: 4.87±2.04 (control): 10.27±1.04 (5 nM, 24 h), 10.35±0.97 (500 nM, 24 h) p = ns) (ACC2 sc: 0.67±0.14 (control): 1.92±0.26, p = ns (5 nM, 24 h), 1.92±0.06, p = ns (500 nM, 24 h)).

**Table 2:** mRNA expression measured by real-time PCR of genes involved in adipocyte lipid homeostasis in differentiated Chub-S7 cells following treatment with Dexamethasone (Dex) alone (a) or in combination with insulin (b) for 24 h.

a	No insulin			b	Insulin (5 nM)		
	No Dex	Dex (5 nM)	Dex (500 nM)		No Dex	Dex (5 nM)	Dex (500 nM)
**SDC1**	362.8±37.3	388.9±34.7	344.9±13.1	**SDC1**	286.5±28.4	233.3±29.5^§^	224.7±30.6
**ACC1**	2.5±0.5	2.4±0.4	1.7±0.8^*^	**ACC1**	1.3±0.4^§^	1.3±0.2^‡^∥	0.9±0.5^*§^
**FAS**	25.6±4.5	33.3±9.2^*^	47.4±7.1^*^	**FAS**	15.6±4.6	29.5±6.4	39.6±10.3^*^
**DGAT1**	2.4±0.37	2.7±0.4	2.5±0.3	**DGAT1**	1.5±0.4	1.9±0.4	1.8±0.3
**GPAT**	0.69±0.14	0.75±0.15	0.57±0.13	**GPAT**	0.42±0.10	0.50±0.13^§^	0.49±0.13
**COP-1**	1.3±0.4	1.5±0.5	1.6±0.5	**COP-1**	1.2±0.3	1.2±0.4	1.2±0.3
**MCD**	0.69±0.15	0.87±0.18	0.90±0.17	**MCD**	0.56±0.20	0.56±0.18	0.76±0.23
**CPT-1**	0.70±0.29	0.81±0.30	0.68±0.30	**CPT-1**	0.79±0.29	0.78±0.27	0.83±0.25
**ACC2**	2.8±0.7	4.3±0.9^*^	7.2±2.2^‡^	**ACC2**	3.3±0.9	5.3±1.1^*^	6.0±1.3^†^
**FAT**	81.4±12.4	87.1±8.1	83.9±11.5	**FAT**	63.6±14.2	65.0±11.4^§^	60.5±12.8
**FABP4**	133.1±16.0	165.2±13.1^*^	178.0±16.8^*^	**FABP4**	89.2±13.6^§^	123.7±15.3^‡§^	120.1±16.6^†§^
**ACS**	3.3±1.1	3.5±1.0	2.0±0.5	**ACS**	1.3±0.5	1.2±0.5^§^	0.81±0.26^‡§^
**AMPKα1**	0.96±0.07	1.1±0.3	1.5±0.2	**AMPKα1**	1.3±0.1	1.2±0.2	1.4±0.2
**PDK4**	2.5±0.6	4.3±1.2	9.1±2.5^‡^	**PDK4**	3.2±0.4	6.0±1.1	10.9±2.0^‡^

Data are presented as arbitrary units±standard error and are the mean of n = 5–7 experiments (* p<0.05, † p<0.01, ‡ p<0.005 vs. no Dex; § p<0.05, ∥ p<0.005 vs. no insulin at same concentration of Dex).

**Figure 2:**
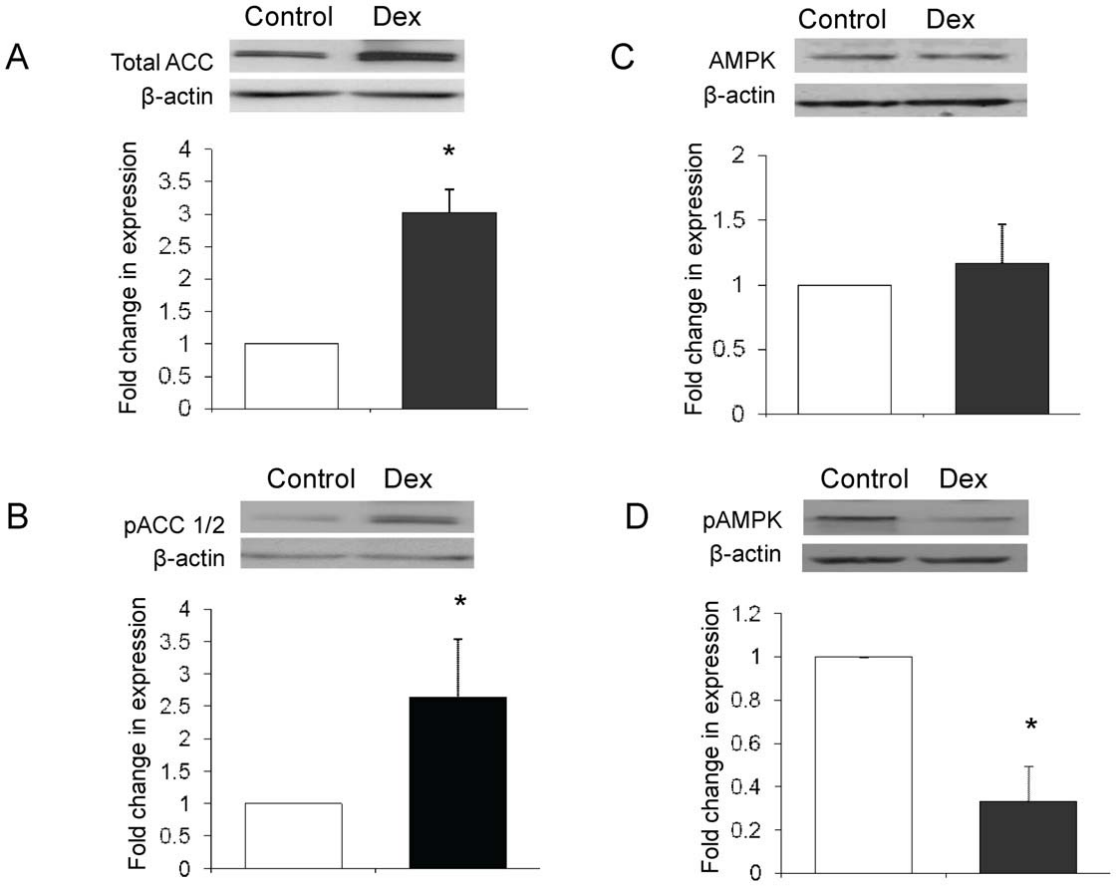
Dexamethasone (Dex, 500 nM, 24 h) increases both total ACC levels (a) and ser-79/218 phosphorylation of ACC (b) in differentiated Chub-S7 cells. Total AMPK levels are not changed (c), but Thr172 phosphorylation is decreased (d). Data are presented as the mean±se fold change compared to untreated cells and quantified relative to β-actin with representative western blots above (n = 3–5 experiments, control open bars, Dex black bars, * p<0.05).

**Figure 3:**
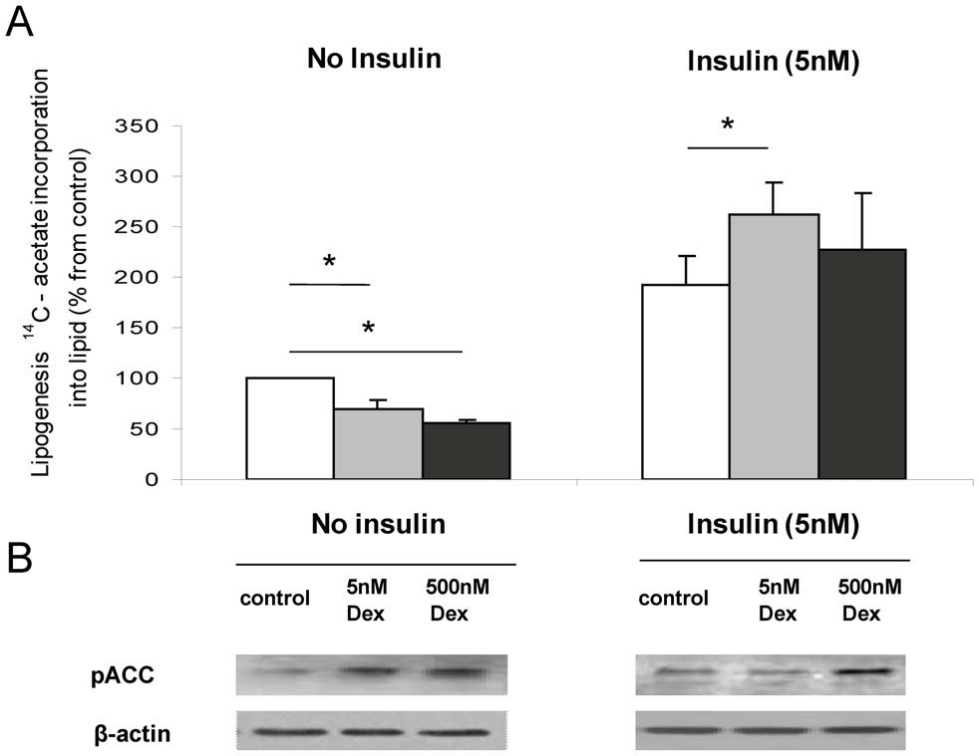
In the absence of insulin, dexamethasone (Dex) decreases lipogenesis as measured by 1-[^14^C]-acetate incorporation into lipid in differentiated Chub-S7 cells in a dose dependent manner (a) and is associated with increasing ser79/218 phosphorylation of ACC (b). Insulin stimulates lipogenesis and the actions of insulin are augmented in the presence of low dose (5 nM), but not high dose (500 nM) Dex (a), where Dex is only able to increase phosphorylation at the higher dose. Control cells are shown in the white bars, low dose dexamethasone treatment (5 nM) grey bars, high dose (500 nM) black bars, * p<0.05) representative western blot is shown.

Whilst Dex (500 nM, 24 h) treatment increased total ACC protein levels (3.0±0.4-fold, p<0.05) in Chub-S7 cells, inactivating ser-79/218 phosphorylation of ACC also increased (2.6±0.9-fold, p<0.05) (figure 2a and b and figure 3b). AMPK protein expression did not change, however phosphorylation of AMPK at thr172 decreased following Dex treatment (figure 2c and d).

**Figure 4:**
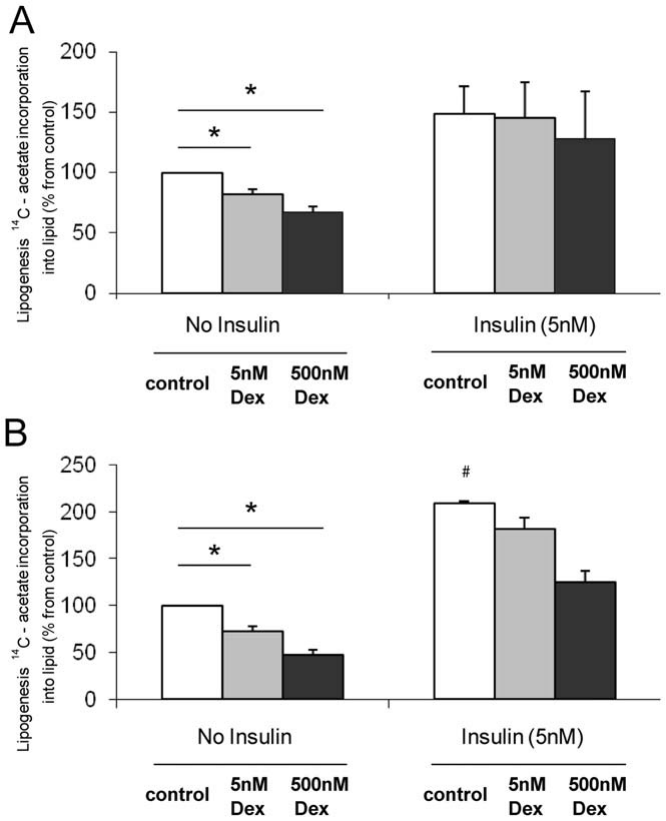
In primary cultures of differentiated subcutaneous (a) and omental (b) pre-adipocytes, Dex decreases lipogenesis in the absence of insulin. Insulin stimulates lipogenesis in both subcutaneous and omental cells (a and b). In omental cells only, Dex impairs the ability of insulin to stimulate lipogenesis (b). (Activity data are expressed relative to untreated cells (no insulin, no Dex) and are the mean±se of n = 3–5 experiments. Control cells are shown in the white bars, low dose dexamethasone treatment (5 nM) grey bars, high dose (500 nM) black bars, * p<0.05 vs. Ctrl and § p<0.05 om, 5 nM insulin vs. sc, 5 nM insulin).

Consistent with increased ser-79/218 phosphorylation of ACC, Dex caused a dose dependent decrease in lipogenesis (100% (control): 70.0±8.2, p<0.005 (5 nM, 24 h), 56.0±3.9%, p<0.01 (500 nM, 24 h)), in Chub-S7 cells (figure 3a and b). These findings were endorsed in primary cultures of differentiated sc and om pre-adipocytes. In cells from both depots, Dex decreased lipogenesis (sc: 81.9±3.9, p<0.005 (5 nM), 67.3±4.8%, p<0.001 (500 nM); om: 72.0±5.4, p<0.005 (5 nM), 46.9±5.7%, p<0.01 (500 nM)) (Figure 4).

### Insulin Stimulated lipogenesis

In Chub-S7 cells low dose Dex (5 nM, 24 h) significantly enhanced the response to insulin (no insulin, no Dex (100%), 192.5±25.3 (5 nM insulin) vs. 248.3±31.4%, (5 nM Dex+5 nM insulin), p<0.05) (figure 3a). Similarly in sc cells, in the presence of insulin (5 nM, 24 h), Dex no longer inhibited lipogenesis and did not impair insulin-stimulated lipogenesis (no insulin, no Dex (100%), 149.0±22.1% (5 nM insulin), 145.5±29.4% (5 nM Dex+5 nM insulin) p = ns) (figure 4a). In contrast, Dex (5 nM and 500 nM, 24 h) decreased lipogenesis in om cells in the presence of insulin, reflecting either a persistence of the direct action of Dex, or its ability to induce insulin resistance and limit insulin-stimulated lipogenesis (no insulin, no Dex (100%), 208.9±1.9 (5 nM insulin), 181.5±11.8%, (5 nM Dex+5 nM insulin) p<0.001) (Figure 4b). In Chub-S7 cells Dex, in the presence of insulin high dose (500 nM, 24 h), but not low (5 nM, 24 h) dose Dex, decreased ACC1 mRNA expression, but increased expression of FAS and ACC2, complete data are presented in table 2a and b. In the primary cells Dex in the presence of insulin increased expression of FAS in sc, but not om, cells although this did not reach significance (FAS sc: 3.84±0.74 (5 nM insulin): 8.81±0.28 (5 nM Dex+5 nM insulin, 24 h), 7.82±0.65 (500 nM Dex+5 nM insulin, 24 h) p = ns). ACC2 expression increased in both sc and om cells (sc: 0.78±0.07 (5 nM insulin): 2.35±0.43, p = ns (5 nM Dex+5 nM insulin, 24 h), 2.45±0.04, p = ns (500 nM Dex+5 nM insulin, 24 h)), (om: 0.08±0.04 (5 nM Dex+5 nM insulin, 24 h): 0.15±0.04, p = ns (5 nM, 24 h), 0.13±0.03, p = ns (500 nM Dex+5 nM insulin, 24 h)) but did not reach significance.

### 11β-HSD1 as a regulator of lipogenesis in human adipose tissue

**Figure 5:**
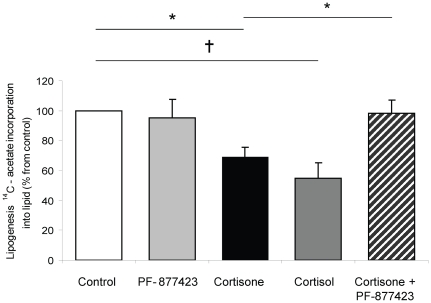
Cortisone (500 nM, 24 h, black) and cortisol (500 nM, 24 h, dark grey) decrease lipogenesis in differentiated Chub-S7 cells. The effects of cortisone are blocked by the 11β-hydroxysteroid dehydrogenase inhibitor, PF877423 (5 µM, 24 h, hashed bar). (Data are expressed relative to untreated cells (white bars) and are the mean±se of n = 3–5 experiments, * p<0.05 and † p<0.01).

We have previously demonstrated the expression of 11β-HSD1 in Chub-S7 cells and characterized its regulation across adipocytes differentiation [17]. 24 h incubation with both cortisol and cortisone decreased basal lipogenesis to a similar extent (100% (control); 65.7±6.2 (cortisone, 500 nM) p<0.001, 50.8±10.9% (cortisol, 500 nM) p<0.005). Incubation with the selective 11β-HSD1 inhibitor (PF-877423, 5 µM) (17) alone was without effect. However, when co-incubated with cortisone (that requires intact 11β-HSD1 activity to generate active cortisol), cortisone-induced repression of lipogenesis was completely abolished (100% (control); 65.7±6.2% (cortisone, 500 nM), 96.4±9.1 (cortisone 500 nM+ PF-877423 5 µM) p<0.01) (figure 5).

### β-oxidation

24 h Dex treatment increased ACC2 and PDK4 mRNA expression (ACC2: 2.8±0.7 (control) 4.3±0.9 (5 nM Dex) p<0.05, 7.2±2.2 (500 nM Dex) p<0.01; PDK4: 2.5±0.6 (control) 4.3±1.2 (5 nM Dex) p = ns, 9.1±2.5 (500 nM Dex) p<0.005) (Table 1a). Dex and insulin treatment, both alone and in combination did not alter β-oxidation, as measured by the conversion of [^3^H]-palmitate to [^3^H]-H_2_O, in differentiated Chub-S7 cells. (100% (control); 102.3±0.6% (5 nM Dex), 104.8±5.5% (500 nM Dex), 105.8±2.2 (5 nM insulin), 102.6±2.6% (5 nM Dex+5 nM insulin), 103.7±2.1% (500 nM Dex+5 nM insulin)

### Fatty acid uptake

In differentiated Chub-S7 cells both low (5 nM, 24 h) and high (500 nM, 24 h) dose Dex increased FABP4 expression (table 1a) and in the absence of insulin increased [^3^H]-palmitate uptake (100% (control); 118.2±5.1% (5 nM Dex), 127.4±6.1% (500 nM Dex) p<0.05).

### Depot Specific Differences

Sc pre-adipocytes differentiated to a greater degree than om pre-adipocytes, with higher mRNA expression of lipid metabolism genes (ACC1 3.2-fold, FAS 10.9-fold, ACC2 17.8-fold, LPL 44.7-fold) and higher 1-[^14^C]-acetate incorporation into lipid compared to om cells (3.8-fold, 564±117 vs. 2154±222 dpm/well, p<0.005). However, lipogenesis in om cells was more responsive to insulin stimulation (100% (control): sc 149±22.14%, om 209±1.86%, p = 0.05) (Figure 4a and b).

## Discussion

The actions of GCs upon adipose tissue are complex, but they are essential for adipocyte differentiation [6]. In this paper, we have performed a detailed characterization of lipid homeostasis in the human pre-adipocyte cell line Chub-S7. Across differentiation, total ACC protein levels increased, although, we were unable to distinguish ACC1 and 2 (due to lack of suitable antibodies) the net effect would be to promote lipid accumulation. Increased ACC1 drives lipogenesis, whilst increased ACC2 will limit β-oxidation and it is this pattern of dynamic lipid flux that we observed across adipocyte differentiation. Consistent with published data [18], insulin was able to stimulate lipogenesis in both undifferentiated and differentiated cells as well as in primary sc and om cultures but failed to regulate β-oxidation. Whilst for the most part the data from the Chub-S7 cell line matched very well with data from primary cultures, it is possible that the transformations used to generate the Chub-S7 cell line could potentially have modified their behaviour and characteristics.

In mature adipocytes, GCs stimulate FFA uptake, *via* lipoprotein lipase activity, as well as driving lipolysis though hormone sensitive lipase [8], [19] and AGTL [20]. In addition, we have previously shown that limiting GC exposure to adipose tissue *in vivo* decreases lipolysis [21]. Dietary TAG is the main source of lipid stored within adipocytes, however, over feeding studies in humans suggest 40% of increases in fat mass are due to *de novo* lipogenesis [22]. In rodent models, GCs inhibit insulin-stimulated *de novo* lipogenesis by decreasing FAS and ACC activity [23], [24]. In the only human study published to date, GC action in isolation was not examined. GCs and insulin, (when compared to insulin alone), increased FAS activity and lipogenesis in sc adipocytes, as measured by glucose incorporation into cellular lipids [25]. In our study, GCs increased ACC2 (but not ACC1) and FAS mRNA expression as well as total ACC protein levels (which may reflect ACC2) but decreased functional lipogenesis in all adipocyte cell models.

Global deletion of ACC1 in rodents results in embryonic lethality [26], but targeted liver-specific deletion, decreases lipogenesis and lipid accumulation without alteration in β-oxidation or glucose homeostasis [27]. In contrast, ACC2 knockout mice have unaltered adipocyte malonyl CoA levels [28] suggesting that it is not involved in lipogenesis. The changes that we have observed in 1-[^14^C]-acetate incorporation into lipid are therefore most likely to reflect regulation of specific ACC1 activity. ACC 1 and 2 are negatively regulated by phosphorylation at Ser-79 and 218 respectively by AMPK [5]. We observed an increase in serine phosphorylation of ACC following GC treatment and this translated to a net decrease in lipogenesis. However, in contrast to observations in hepatocytes where GCs increase AMPK expression [29] this was unchanged in Chub-S7 cells, sc and om human primary cultures (data not shown). Furthermore, activating phosphorylation of AMPK at Thr172 was decreased by GC treatment suggesting that regulation of ACC1 through serine phosphorylation is likely to be independent of AMPK in human adipose tissue. Our data are in agreement with studies on adipocytes differentiated from mesenchymal stem cells where Dex did not alter gene or protein expression, but decreased AMPK activity [30] and with obervations in adipose tissue fom patients with Cushing's syndrome, where AMPK phosporylation is decreased [31].

In Chub-S7 cells, Dex augmented insulin-stimulated lipogenesis and this is in agreement with our previous studies [14] as well as others [32] that have shown enhanced activation of the insulin signalling cascade and glucose uptake following pre-treatment of adipocytes with GC. In sc and om cells in this study, the incremental response to insulin stimulation was similar with and without low dose Dex and suggests that this dose (5 nM) is not sufficient to cause insulin resistance. At the highest dose (500 nM) in om, but not sc cells, Dex in combination with insulin decreased lipogenesis. Although these changes were not matched by the expected pattern of changes in mRNA expression, it is possible that this may reflect the complex post-translational regulation of lipogenesis and it is entirely plausible that there may exist a depot-specificity of regulation. In addition, extrapolating these observations to the *in vivo* situation is complex and represents a balance of ‘direct’ GC actions to decrease lipogenesis as well as any potential action to cause insulin resistance and abrogate the effect of insulin treatment.

These observations do raise the intriguing possibility of differential nutritional regulation of lipogenesis. In the fasting state, low insulin levels and high endogenous GC levels will stimulate lipolysis and simultaneously switch off lipogenesis though serine phosphorylation of ACC1, decreasing fuel storage and increasing FFA availability for other more metabolically active tissues. Conversely, in the fed state, insulin levels are high, and here insulin and GC may act together to promote lipid storage. However, we must exert a note of caution as extrapolating from *in vitro* models to *in vivo* observations is fraught with difficulty and these hypotheses will need to be tested in dedicated, well designed clinical studies.

Whilst obesity *per se* is detrimental to health, regional distribution of adipose tissue is crucially important. Intra-abdominal, as opposed to sc adipose deposition, correlates with insulin resistance [33] and increased cardiovascular morbidity and mortality [34]. The molecular mechanisms that underpin depot specific differences in fat accumulation are therefore of great interest. This study used differentiated pre-adipocytes from paired sc and om depots and as sc cell in culture differentiate to a greater degree comparisons of hormone responsiveness between cell types is difficult. Differences in insulin action between adipose tissue depots have been described [35]–[38]. Interestingly, despite a lower level of differentiation, lipogenesis in om cells responded more dramatically to insulin treatment, however, a dedicated study in a more suitable model is required.

GC availability and action not only depend upon circulating levels, but also on the tissue specific intracellular activation or inactivation by 11β-HSD. *In vitro*, rodent and clinical data have endorsed 11β-HSD1 as a metabolic therapeutic target [39]– and inhibitor studies including phase II studies in man are now published [13]. Improvements in lipid profiles as well as insulin sensitivity have been demonstrated for a number of compounds, but the precise mechanisms by which they convey their metabolic benefit remains to be confirmed. In skeletal muscle, selective 11β-HSD1 inhibitors alter lipid metabolism decreasing FFA availability [42] and this may underpin their ability to cause insulin sensitization through a putative impact to limit protein kinase C activation [43]. In adipose tissue we have previously demonstrated that 11β-HSD1 inhibition decreases lipolysis [21] and have now shown that these compounds can enhance lipogenesis. Taken together, these observations suggest that decreased lipid mobilization may represent a potential mechanism by which these compounds improve metabolic phenotype.

In conclusion, we have demonstrated that GCs have potent actions upon lipid homeostasis and also interact with insulin in human adipocyte cell lines and primary cultures. Manipulation of GC availability through selective 11β-HSD1 inhibition has therapeutic potential by altering lipid homeostasis in key metabolic target tissues and this may underpin their putative action as insulin sensitizers.
